# Engineering Intrinsically Zirconium‐89 Radiolabeled Self‐Destructing Mesoporous Silica Nanostructures for In Vivo Biodistribution and Tumor Targeting Studies

**DOI:** 10.1002/advs.201600122

**Published:** 2016-05-27

**Authors:** Shreya Goel, Feng Chen, Shijie Luan, Hector F. Valdovinos, Sixiang Shi, Stephen A. Graves, Fanrong Ai, Todd E. Barnhart, Charles P. Theuer, Weibo Cai

**Affiliations:** ^1^ Materials Science ProgramUniversity of Wisconsin–MadisonMadisonWI53705USA; ^2^ Department of RadiologyUniversity of Wisconsin–MadisonMadisonWI53705USA; ^3^ School of PharmacyTemple UniversityPhiladelphiaPA19140USA; ^4^ Department of Medical PhysicsUniversity of Wisconsin–MadisonMadisonWI53705USA; ^5^ TRACON Pharmaceuticals IncSan DiegoCA92122USA; ^6^ University of Wisconsin Carbone Cancer CentreMadisonWI53705USA

**Keywords:** biodegradable mesoporous silica nanoparticles, intrinsic radiolabeling, positron emission tomography (PET) imaging, vasculature targeting

## Abstract

A systematic study of in vitro and in vivo behavior of biodegradable mesoporous silica nanoparticles (bMSNs), designed to carry multiple cargos (both small and macromolecular drugs) and subsequently self‐destruct following release of their payloads, is presented. Complete degradation of bMSNs is seen within 21 d of incubation in simulated body fluid. The as‐synthesized bMSNs are intrinsically radiolabeled with oxophilic zirconium‐89 (^89^Zr, *t*_1/2_ = 78.4 h) radionuclide to track their in vivo pharmacokinetics via positron emission tomography imaging. Rapid and persistent CD105 specific tumor vasculature targeting is successfully demonstrated in murine model of metastatic breast cancer by using TRC105 (an anti‐CD105 antibody)‐conjugated bMSNs. This study serves to illustrate a simple, versatile, and readily tunable approach to potentially overcome the current challenges facing nanomedicine and further the goals of personalized nanotheranostics.

## 1 Introduction

Inorganic nanomaterials have rapidly gained momentum in nanomedicine due to their unique physicochemical properties and partly due to their facile synthesis, biofunctionalization, and ease of loading a wide range of therapeutics.^1^ However, only a few have advanced beyond preclinical studies and even fewer have been approved by the U.S. Food and Drug Administration (FDA) for clinical purposes.^2^, ^3^ An ideal theranostic nanoparticle is one which is safe and non‐immunogenic, can rapidly and specifically accumulate in the diseased tissue, map the biomedical/morphological status of the tissue, effectively deliver the therapy, and be degraded or cleared from the body in a reasonable time period.^1^ In this regard, biodegradable and renally clearable nanoparticles have emerged as frontrunners.^4^ While renal clearable nanomaterials (with a hydrodynamic diameter typically less than the glomerular threshold limit of ≤7 nm) alleviate the long‐term toxicity concerns, great challenges still exist in engineering ultra‐small multifunctional nanoparticles with tunable blood circulation half‐life, specific, enhanced, and persistent tumor accumulation, etc.^4^ Thus, a novel nanoplatform with suitable blood residence time and tumor‐specific targeting ability in vivo, and that can self‐destruct into small fragments to facilitate clearance might hold greater promise for future clinical translation.

Majority of previous reports on nanoparticle mediated drug delivery have been optimized only for small‐molecule drugs, thereby highlighting the urgent need for development of nanocarriers for potent biomacromolecular drugs, a rapidly developing area in cancer therapeutics.^5^, ^6^, ^7^, ^8^ Despite the therapeutic activity of a single bio‐macromolecular drug (e.g., small proteins, antibodies, nucleic acids, etc.) being equivalent to about 10^6^–10^8^ of that a conventional small molecule anti‐cancer agent (e.g., doxorubicin), issues like inefficient delivery to the target site and inability to cross the tumor cell membrane have severely limited their clinical applications.^5^ Mesoporous silica nanoparticles (MSNs), due to their large specific surface area, pore volume, and well‐defined and easily tunable porous structure, have become one of the most intensively studied nanomaterials and have evolved spectacularly in the last two decades.^9^, ^10^ Silicon is a well‐known trace element in the human body and silica (i.e., SiO_2_) has been categorized as a “generally recognized as safe” (GRAS) by the FDA (ID Code: 14808‐60‐7).^11^ Moreover, C dots are the only renal clearable inorganic nanoparticles that have gained Investigational New Drug (IND) approval by the FDA for first‐in‐human clinical trials (clinicaltrials.gov; ID: NCT01266096, NCT02106598).^2^, ^12^, ^13^ However, suboptimal in vivo pharmacokinetics of most reported MSNs, namely, inefficient tumor targeting and rapid sequestration by the reticuloendothelial system (RES, e.g., liver and spleen), as well as slow degradation and excretion have impeded their translational ability.^14^ To address these challenges, we propose an improved design for the silica nanoconjugates, wherein they can (1) entrap large molecular drugs, (2) self‐destruct over time, without the aid of any external agent or stimulus, (3) rapidly and specifically accumulate in tumor tissues in vivo, and (4) be tracked over a long period of time using intrinsically labeled zirconium‐89 (^89^Zr, *t*_1/2_ = 78.4 h) and highly sensitive and quantitative positron emission tomography (PET) imaging.

Dendritic, biodegradable mesoporous silica nanoparticles (bMSNs) were synthesized, with a centrally radiating, two‐tiered porous network. The large pores (≈12 nm) served to load macromolecular cargo, as well as accelerate the degradation of the nanoparticles. The abundant deprotonated silanol (Si—O^−^) groups in bMSN were harnessed for chelator‐free labeling of oxophilic position emitting isotope (e.g., ^89^Zr) for tracing the in vivo biodistribution. The design of the nanoconjugates was further optimized for effective enhanced tumor vasculature targeting in 4T1 breast cancer murine models.

## 2 Results and Discussion

### 2.1 Synthesis and Characterization of bMSNs

**Figure 1:**
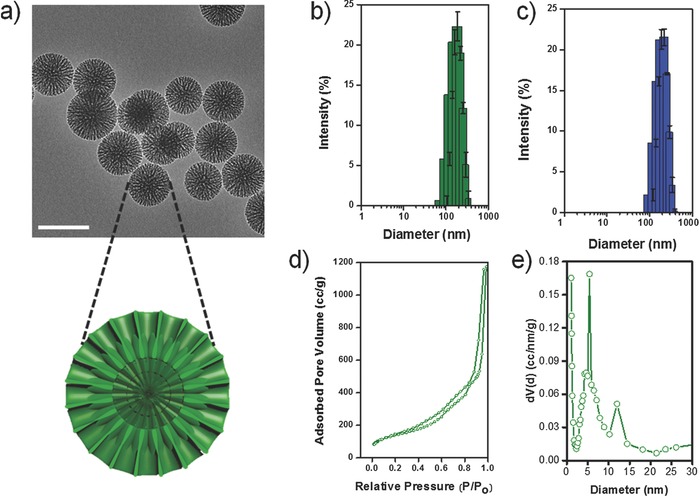
Physicochemical characterization of bMSN. a) TEM image shows an average particle size ≈160 nm. (Scale bar: 200 nm) Outset schematic depicts the cross section of bMSN showing a two‐tiered porous structure. The inner pores measure ≈5.4 nm that continue into outer wider channels ≈12 nm in diameter. Hydrodynamic size distribution of b) bMSN and c) bMSN‐PEG_5k_‐TRC105. d) Nitrogen sorption isotherms and e) bimodal pore size distribution of calcined bMSNs.

Uniformly sized bMSNs with hierarchical dendritic networks were synthesized using a modified oil−water bi‐phase stratification reaction system as reported in literature (see the Experimental Section for details).^15^ Representative transmission electron microscopy (TEM) images (**Figure** **1**a) indicate monodispersed mesoporous nanopaticles, ≈162.9 ± 6.4 nm in diameter bearing a spoke‐like radiating, rigid meso‐channeled internal structure. The results were consistent with the dynamic light scattering (DLS) measurements which showed bMSNs with a slightly larger hydrodynamic diameter of 190.1 ± 16.9 nm (Figure 1b). Furthermore, the concentration of bMSNs (number of nanoparticles mL^−1^) was determined using the nanoparticle tracking analysis (NTA) method on a NanoSight instrument (Video S1, Supporting Information). Results indicated a highly homogenous population with the estimated value of ≈8.97 × 10^11^ nanoparticles mL^−1^ or 3.59 × 10^14^ g^−1^ (Figure S1, Supporting Information). These values also corroborated our theoretically calculated number of 9.68 × 10^14^ bMSNs g^−1^ (detailed calculations shown in Section S.1, Supporting Information). Based on the Zhuravlev model,^16^ the concentration of silanol groups was estimated to be ≈1 × 10^7^ per nanoparticle (Section S.2, Supporting Information).

The Brunauer–Emmett–Teller (BET) measurements indicated a high surface area ≈741.92 m^2^ g^−1^ and a large pore volume (1.84 cm^3^ g^−1^). As apparent from the nitrogen sorption isotherms (Figure 1d), as‐synthesized bMSNs demonstrate a broad pore size distribution, with the appearance of hysteresis and capillary condensation steps at relative pressures 0.2 < *P*/*P*_0_ < 0.8. The radial spoke‐like, two‐level pore structure appeared to possess a central core of densely packed channels (pore size: ≈5.4 nm) which continued into an outer connected ring of wider channels (pore size: ≈12.0 nm), seen clearly in Figure 1a. The bi‐modal pore size distribution was further confirmed by Barrett–Joyner–Halenda (BJH) analysis (Figure 1e). In addition, we also observed that using our simplified one‐pot synthetic protocol, the pore size could be easily tailored by simply altering the precursor (i.e., tetraethyl orthosilicate [TEOS]) to organic solvent (i.e., cyclohexane) ratio and reaction time; thus, bypassing the requirement of different organic solvents as reported previously.^15^ For example, by reducing the amount of TEOS precursor used (ratio: 1 v/v%) and extending the reaction time to 70 h, bMSNs with particle size of ≈285 nm and pore size of ≈17 nm were obtained (Figure S2, Supporting Information).

### 2.2 Degradation Study of Silica Nanoparticles (SNs)

**Figure 2:**
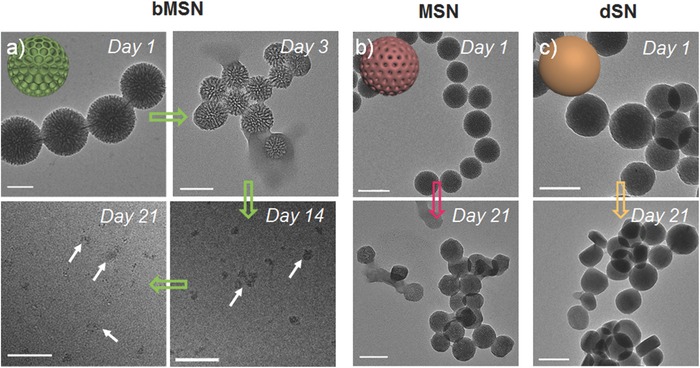
In vitro self‐destruction of SNs. Serial TEM images of biodegradation behavior of a) bMSNs, b) MSNs, and c) dSNs, in a continuously stirred SBF environment at 37 °C. All scale bars are 100 nm.

**Figure 3:**
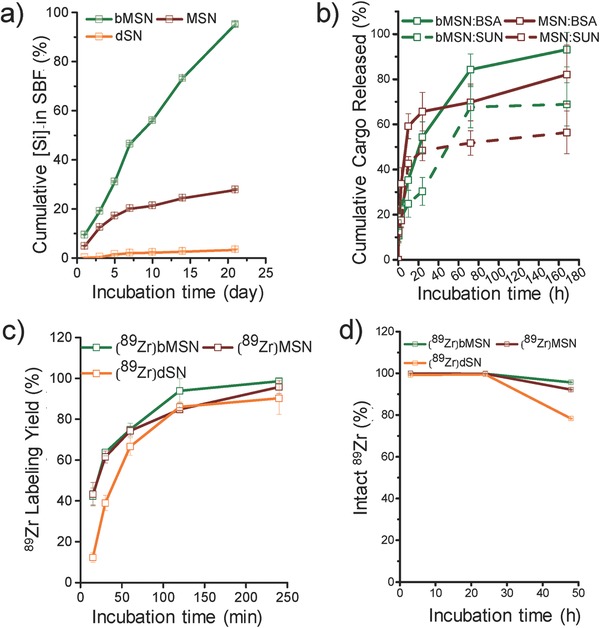
Systematic in vitro study. a) MP‐AES analysis of [Si] accumulated in SBF (at 37 °C, pH ≈5). b) Cumulative release of SUN and BSA (in SBF at 37 °C, pH ≈5). c) ^89^Zr labeling yields as a function of incubation time at 75 °C, pH 7–8. d) Stability of ^89^Zr labeled SNs, in mouse serum at 37 °C over 7 d (*n* = 3).

Rapid sequestration of nanoparticles with a hydrodynamic size larger than ≈10 nm by the macrophages, and subsequent non‐specific and prolonged residence of these phagocytosed nanoparticles in non‐targeted organs (such as liver and spleen) has challenged the effectiveness of nanotheranostics.^1^ In this regard, self‐destructing silica nanoparticles which degrade into non‐toxic and non‐immunogenic byproducts over time, can be a promising platform for nanoparticle based in vivo imaging and drug delivery. To test the degradability of bMSNs, as‐synthesized nanoparticles were incubated in simulated body fluid (SBF) at 37 °C for a period of 21 d with constant stirring. It is well known that upon intracellular uptake, nanoparticles are routed to lysosome organelles, which are primary sites of intracellular sequestration and degradation of foreign particles and pathogens.^17^ Accordingly, the pH of the soaking solution was tuned to 4–5 to mimic the natural lysosomal environment. Aliquots were drawn at frequent intervals and centrifuged at high speed. The pellet was observed using TEM and the supernatant was analyzed with microwave plasma – atomic emission spectroscopy (MP‐AES) to assess the Si concentration. Distinct changes in nanoparticle morphology could be observed within 1 d post‐incubation, when the outer edges began to erode into rougher surfaces (**Figure** **2**a, Day 1). The degradation accelerated thereafter and subsequent signs of obvious degradation could be seen at day 3 (Figure 2a, Day 3). ≈19.0 ± 0.1% of the initial Si ion concentration was released into the SBF solution within 3 d of incubation. Erosion progressed inward from the outer edges, resulting in smaller and irregularly shaped nanoparticles and fragments (Figure 2a, Day 14). MP‐AES analysis corroborated these observations. With further degradation, the solution turned almost transparent from an initial milky white to completely transparent by day 21 (Figure S3c, Supporting Information). Only debris like silica fragments could be seen under the TEM (Figure 2a, Day 21) and almost complete breakdown resulted in ≈95.2 ± 0.90% Si leaching into the solution, in the form of silicic acid (**Figure** **3**a).

To evaluate the influence of structure and pore size on degradability, two controls were used: solid or dense silica nanoparticles (dSNs; with no mesopores) and regular MSNs with a smaller pore size (2–3 nm). While bMSNs show a radially arranged porous network, dSNs have minimal porous structure and MSNs are composed of irregularly oriented mesochannels (Figure S3a,b, Supporting Information). As expected, both dSNs and MSNs showed much lower rates of degradation when compared to bMSNs. Even after 21 d of incubation in SBF, dSNs failed to show any appreciable change in morphology (Figure 2c, Day 21) or presence of Si ions in SBF (Figure 3a, orange line). MSNs displayed mild signs of degradation with somewhat distorted morphology and collapsed porous network after 21 d in SBF (Figure 2b, Day 21) and only ≈28% of the initial Si ion concentration was released at the end of the study period (Figure 3a, maroon line).

The much more rapid degradation rate for bMSNs can be explained on the basis of their higher surface areas and lower degree of cross‐linking of the silicate skeleton. The large pore size and hierarchically organized pores display a much larger surface area, as demonstrated earlier, which has been shown to enhance the degradation rate.^18^ In addition, *Q* ^3^/*Q* ^4^ ratio for dendritic bMSNs has been demonstrated to be ≈0.4–0.5,^15^ much smaller than that for MSNs^19^ and dSNs.^20^ The much lower degree of cross‐linking between the silicate groups in bMSN may stem from the formation of dendritic porous channels resulting from the gentle condensation of silicates at the oil–water interface via the biphase stratification approach. In contrast rapid disorganized condensation of MSN porous network in the Stöber process likely gives rise to a highly cross‐linked silicate framework, which in turn may impede the degradation process. Further studies will be needed to study the degradation mechanism in detail. These results demonstrate that a hierarchical porous structure and larger pore diameter enhance the degradability of SNs by several folds, indicating the potential of bMSNs as a promising theranostic nanoplatform. Since, the physiological milieu is more complex, we believe that actual degradation of the nanoparticles should be more rapid in vivo, possibly aided by lysosomal enzymes. Ongoing studies aim to trace and improve upon the degradation process in vivo and decipher the exact mechanism. It is also worth mentioning that this time line of degradation of nanoparticles complements the purpose of drug delivery and imaging, where the nanocarrier is expected to degrade quickly enough to prevent any toxic reaction resulting from non‐specific accumulation, yet slow enough to prevent premature release of cargo during circulation. This is especially important for nanosystems with long blood circulation half‐life. Hence, proper control over the degradation time and rate is essential for designing a clinically relevant nanovehicle. Extensive in‐depth studies are required in the future to carefully tailor the nanoparticle design and functionalization to develop an ideal, clinically translatable drug delivery vehicle.

### 2.3 Cargo Loading and Release

Tumor cell heterogeneity and its implication for drug resistance have been the driving force for combination chemotherapy.^21^ MSNs with their high surface area‐to‐volume ratios and easily tailorable chemistry provide an ideal platform to concurrently deliver multiple types of drugs to synergistically improve the efficacy of cancer therapeutics.^10^ Accordingly, to evaluate the effect of pore size and structure on cargo loading capacity, as‐synthesized SNs were suspended in a dimethyl sulfoxide (DMSO) solution of small molecule, hydrophobic anti‐angiogenesis drug (i.e., sunitinib malate [SUN]). Absence of a porous network resulted in minimal drug loading in dSNs, which showed only 8.4% loading efficiency. MSNs and bMSNs on the other hand, demonstrated high loading capacity, with up to 155.09 and 295.95 mg SUN loaded per g of nanoparticles, respectively (Table S2, Supporting Information). The large pore size of bMSNs provides an opportunity to load biomacromolecular therapeutics such as small proteins and even antibodies. Bovine serum albumin (BSA) (molecular weight ≈66.5 kDa) was used as a model protein in this study to demonstrate the dual loading capacity of SNs. DLS measurements indicated the hydrodynamic size of BSA in PBS to be ≈8.63 nm; PDI 0.21. SUN loaded nanoparticles were suspended in a 2 mg mL^−1^ PBS solution of BSA and gently mixed overnight. As expected, solid dSNs showed negligible encapsulation of the protein (≈1.5%). The effect of pore size on protein encapsulation was apparent. While small molecule drugs could be loaded at similar efficiency, distinct differences could be observed in protein loading capacities of the two types of porous nanostructures. ≈589.28 mg of BSA was encapsulated per g of bMSNs while only 250.0 mg could be loaded per g of MSN (Table S3, Supporting Information). Literature indicates that BSA assumes a prolate ellipsoidal structure hydrodynamically, with a longer and shorter axis.^22^ The lower but not zero “loading” of BSA into MSNs may result from wedging of the protein molecules into the smaller pore entrances through their shorter axes. The much larger hydrodynamic diameters compared to the small pore size (≈2–3 nm) of MSN rules out any real BSA loading inside the nanoparticles. SUN loading did not appreciably affect the loading of BSA, further attesting the efficacy of bMSNs to serve as nanocarriers for multiple cargo types and highlighting their potential for use in combination chemotherapy.

The release of SUN and BSA from bMSNs and MSNs was then studied at 37 °C in SBF (pH 5.0) to mimic the tumor microenvironment. Aliquots were drawn at regular time points and centrifuged to separate solid silica nanoparticles with entrapped SUN and BSA. The concentration of the released SUN and BSA was determined by measuring the UV–vis absorption profiles of the supernatant at 460 and 280 nm, respectively. bMSNs and MSNs show distinctly different release profiles for both small molecule drug and larger protein (Figure 3b), which can be attributed to the differences in their mesostructures. MSNs with irregular mesoporous network of channels show an early burst release for both SUN and BSA within 10 h post‐incubation (42.74 ± 2.98% and 59.22 ± 5.54%, respectively). The quick release of SUN can be attributed to the diffusion of the drug loaded inside the MSN pores, climaxing at ≈56% cumulative release, upon reaching equilibrium. The rapid shedding of BSA was attributed to the dynamic nature of the in vitro experiment, since loosely wedged BSA molecules in the MSN pores could be easily freed in solution under shaking conditions. bMSNs, on the other hand, showed a multistage release for both SUN and BSA. Slow and steady release up to 24 h is followed by a sharp spike around day 3 (≈67.67 ± 9.12% SUN and 83.31 ± 6.89% BSA released) which coincides with accelerated fragmentation of bMSNs, indicating that degradability of bMSNs plays an important role in enhanced release of the loaded cargo. While traditional small pore MSNs rely only on equilibrium‐based diffusion for release of the cargo, degradation of bMSNs provides greater opportunity for complete cargo release. Cumulative release of encapsulated drugs was found to be higher for bMSN (68.9 ± 9.57% SUN and 93.13 ± 7.67% BSA), when compared to MSN, further attesting the superiority of the degradable silica nanostructures. In addition to the enhanced release, the currently reported release profile is also better suited for longer circulating nanoparticles. A carefully tuned, slower release profile prevents premature shedding of cargo before the nanoconjugates have time to reach their target. Compared to the traditionally reported initial‐burst‐release behavior, a multi‐stage release mechanism would potentially minimize off‐target toxicity. Moreover, the larger pores of bMSNs encapsulate larger amount of cargo and act as a protective support from the exogenous proteins and proteolytic attack in vivo, thereby enhancing the therapeutic index of the drugs multifold.

### 2.4 Intrinsic ^89^Zr Labeling of Silica Nanoparticles

We recently reported a novel application of mesoporous silica nanomaterials in intrinsic chelation of specific oxophilic radionuclides such as ^89^Zr.^23^ ^89^Zr has an optimal half‐life (*t*_1/2_ = 78.4 h) and relatively low positron energy (*β* ^+^_avg_ = 395.5 keV), making it suitable for long‐term in vivo tracking of nanoparticles, low uptake in the background tissue, and optimal dosimetry.^24^ For these reasons, ^89^Zr based radiopharmaceuticals are being actively translated to the clinic, with several clinical trials underway.^25^ The widely used protocol for ^89^Zr labeling involves a hexadentate chelator, desferrioxamine B (DFO). However, studies using DFO labeled tracers have still shown a significant bone uptake (after a week post‐injection (p.i.)), indicative of the detached ^89^Zr, a well‐known osteophile.^26^, ^27^ Intrinsic labeling involving a carefully selected nanoplatform/radioisotope pair, can potentially overcome the problems associated with chelator instability and tedious chemistries for chelator functionalization.^28^

In the present study, silica nanoparticles were radiolabeled as described earlier.^23^ Briefly, SNs were incubated with 111 MBq of ^89^Zr (specific activity ≈20 GBq μmol^−1^) in 0.1 m HEPES (4‐(2‐hydroxyethyl)‐1‐piperazineethanesulfonic acid) buffer, pH 8 for 4 h at 75 °C, with constant vigorous shaking. Rapid and high‐yield labeling was observed for all SNs. Comparable ^89^Zr labeling yields were observed for bMSNs (98.6%) and MSNs (95.8%) and slightly lower for dSNs (90.3%) (Figure 3c). However, serum stability tests are essential to assess the suitability of radiolabeled nanoparticles for in vivo applications. As evident from Figure 3d, majority of the ^89^Zr remained stably bound to bMSNs (95.60 ± 0.01%) and MSNs (92.13 ± 0.02%) up to 48 h post‐incubation in mouse serum. In comparison, ^89^Zr labeled dSNs showed much poorer stability, with only 52.84 ± 0.03% ^89^Zr remaining stably chelated. High fidelity of the radiolabel to bMSNs can be attributed to the well‐defined radial network of channels, which allows easy access to ^89^Zr ions into the interior recesses. Due to a large surface area, the core of the nanoparticle is expected to possess more neighboring deprotonated —Si—O^−^ groups which can stably chelate the oxophilic ^89^Zr, as compared to that on the surface. We believe that a much higher surface area of bMSN (≈741 m^2^ g^−1^) stemming from hierarchically ordered large pore channels, compared to MSN (≈238 m^2^ g^−1^),^29^ ensures the availability of greater number of deprotonated —Si—O^−^ groups available for more stable chelation of ^89^Zr. dSNs, on the other hand, possess little to no porous structure, keeping majority of the chelated ^89^Zr ions to the surface with fewer —Si—O^−^ groups. The coordination is expected to be weaker and hence ^89^Zr can easily dissociate from the parent dSNs over time. Encouraged by these results, ^89^ Zr‐labeled bMSNs, were subsequently functionalized for in vivo studies.

### 2.5 Synthesis and Characterization of [^89^Zr]bMSN‐PEG_5k_‐TRC105

**Scheme 1:**
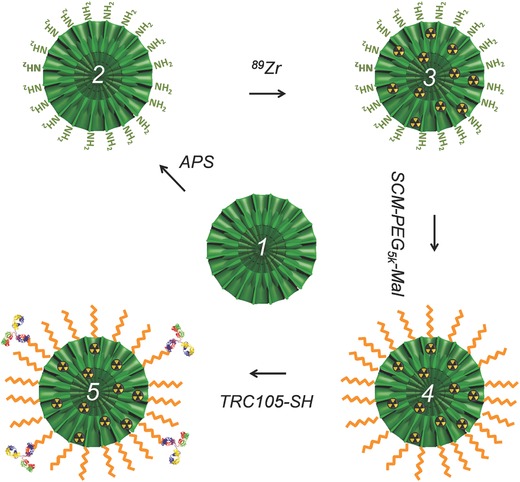
Schematic depiction of step‐by‐step synthesis of [^89^Zr]bMSN‐PEG_5k_‐TRC105. Uniform bMSNs (***1***) were modified with —NH_2_ groups to form bMSN‐NH_2_ (***2***), followed by direct chelator‐free labeling with oxophilic ^89^Zr to form [^89^Zr]bMSN‐NH_2_ (**3**). Then (**3**) was PEGylated twice to yield [^89^Zr]bMSN‐PEG_5k_‐Mal (**4**) which was subsequently conjugated with TRC105‐SH to obtain the final nanoconstructs, [^89^Zr]bMSN‐PEG_5k_‐TRC105 (***5***).

Prior to in vivo applications, as‐synthesized bMSNs were surface modified to impart optimal circulation time and tumor targeting capability, followed by systematic and thorough characterization. **Scheme** **1** depicts the major steps involved in the synthesis of [^89^Zr]bMSN‐PEG_5k_‐TRC105. bMSNs were first functionalized with amine groups (—NH_2_) to aid in subsequent surface conjugations for in vivo studies. bMSN‐NH_2_ was then radiolabeled in 0.1 m HEPES buffer, pH 8 for 2 h at 75 °C. Presence of —NH_2_ groups on bMSN did not appreciably affect the radiolabeling yield (≈94.7% within 2 h post‐incubation). The role of deprotonated silanol groups in ^89^Zr chelation was also confirmed by incubating bMSN‐NH_2_ in 0.1 m HEPES buffer, with the pH adjusted to 2. As expected, the radiolabeling yield was drastically reduced due to complete protonation of bMSNs under highly acidic conditions (Table S4, Supporting Information). Further, [^89^Zr]bMSNs‐NH_2_ was camouflaged with SCM‐PEG_5k_‐Mal, in order to make them more bioactive and immune evasive for in vivo studies. Effective PEGylation is critical for longer circulation of nanoparticles and must be carefully tailored for each individual nanosystem. Succinimidyl carboxymethyl (SCM) is an NHS ester routinely used for PEGylation of primary amine bearing nanoparticles via amide linkage. However, it hydrolyzes rapidly in aqueous environments (0.75 min, pH 8). In the present study, two‐step PEGylation was found to be more effective than a single step with same PEG amount and longer duration. The nanoconjugates demonstrated greater stability (at least two weeks in PBS) and longer circulation in vivo (data not shown).

Next, tumor targeting ability was conferred to the nanoconjugates by attachment of an anti‐CD105 antibody, i.e., TRC105, to the PEGylated samples. The antibody molecules were derivatized with thiol (—SH) groups by reaction with Traut's reagent. About three thiol moieties per TRC105 were found to be optimal for nanoparticle conjugation. The conjugation of TRC105 to [^89^Zr]bMSN‐PEG_5k_‐Mal was found to acutely depend on the reaction ratio, incubation time, and the number of reactive thiol groups on each antibody and had profound effect on in vivo biodistribution and targeting efficacy. Optimal results were obtained with the current protocol (details in the Experimental Section). Systematic calculations based on Bradford Assay, indicated that each bMSN has about ≈44 TRC105 molecules attached for an effective active targeting (Section S.3, Supporting Information). It is worth noting here that this number corroborates data from a previous report, where high (10^4^ antibodies/nanoparticle) and low (≈15 antibodies/nanoparticle) ligand densities were shown to adversely affect the nanoparticle–receptor/antigen interactions due to crowding effects, and orientation and unfolding issues, respectively.^30^ Our ongoing studies aim to assess the effects of ligand density on in vivo nanoparticle pharmacokinetics and tumor targeting ability.

Success of each step of the conjugation protocol was confirmed by the concurrently changing zeta potential values (Table S5, Supporting Information). As‐synthesized bMSNs were negatively charged in PBS (−48.37 ± 0.31 mV) as expected due to deprotonated silanol groups at pH > 2. [^89^Zr]bMSN‐PEG_5k_‐TRC105 demonstrated a final zeta potential of −0.16 ± 0.06 mV. This near neutral zeta potential of the nanoconjugates is advantageous since neutrally charged nanoparticles have been shown to circulate longer in the blood than their charged counterparts.^31^

### 2.6 In Vivo Biodistribution and Tumor Vasculature Targeting with [^89^Zr]bMSN‐PEG_5k_‐TRC105

**Figure 4:**
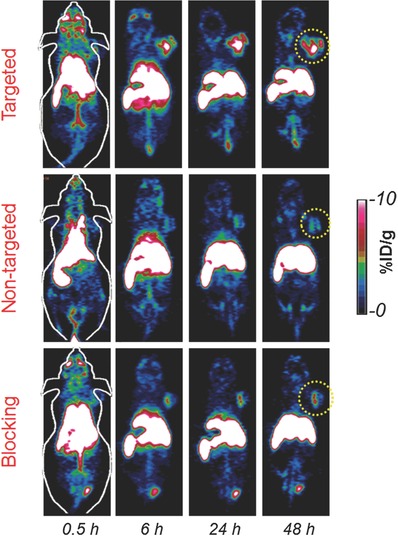
In vivo tumor vasculature targeting. Representative tumor‐bearing coronal slices from serial PET scans. Targeted group: [^89^Zr]bMSN‐PEG_5k_‐TRC105; non‐targeted group: [^89^Zr]bMSN‐PEG_5k_ and blocking group: [^89^Zr]bMSN‐PEG_5k_‐TRC105 (with a pre‐injected blocking dose of TRC105). Yellow circles indicate the location of 4T1 breast tumor.

Angiogenesis plays a key role in tumor growth and metastasis and represents one of the most promising hallmarks for cancer diagnosis and treatment.^32^ CD105 or endoglin receptor, highly overexpressed on angiogenic endothelial cells and upregulated under hypoxic conditions, is a prime target for tumor imaging, prognosis, and anti‐angiogenic therapy in a variety of cancers including breast, colon, brain, and prostate.^33^ TRC105 (a human/murine chimeric IgG1) is a high avidity CD105‐targeted antibody, currently under multiple Phase II clinical trials.^34^ To assess the CD105 targeting of [^89^Zr]bMSN‐PEG_5k_‐TRC105, ≈2.8 × 10^10^ bMSN nanoconjugates were injected intravenously in 4T1 tumor bearing mice (≈14 mg kg^−1^) and serial PET scans were performed 0.5, 6, 24, and 48 h p.i. Mice were randomized before injections and divided into three groups (*n* = 3 per group) designated as targeted, non‐targeted, and blocking. Tumor‐bearing coronal slices are shown in **Figure** **4**. Region‐of‐interest (ROI) analysis was also performed and the time–activity curves for the major sites of nanoparticle uptake can be seen in Figure S5 (Supporting Information).

**Figure 5:**
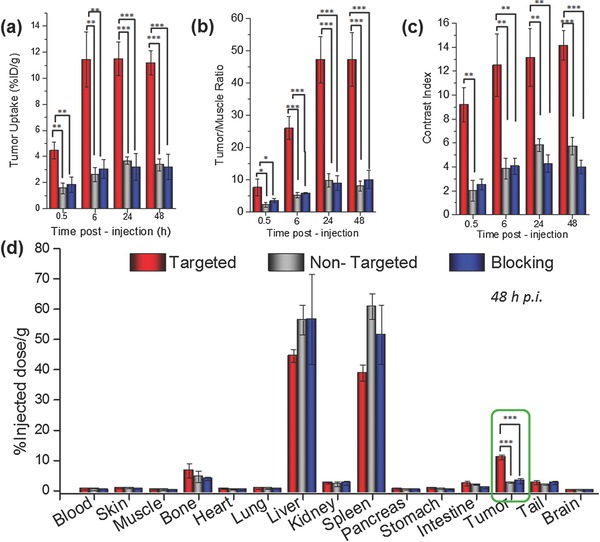
Quantitative region‐of‐interest (ROI) analysis and ex vivo biodistribution. Temporal variation of a) absolute tumor uptake, e) tumor‐to‐muscle ratio, and f) contrast index, for the targeted, non‐targeted, and blocking groups. d) Ex vivo biodistribution data, 48 h p.i. (*n* = 3). The differences in tumor uptake between the targeted group and two control groups were statistically very significant (*P < 0.05, **P < 0.001, ***P < 0.001).

Rapid and persistent uptake of nanoparticles was observed in the targeted group (4.5 ± 0.6%ID g^−1^) as soon as 0.5 h p.i., which increased to 11.4 ± 2.1%ID g^−1^, 6 h p.i., remaining constant thereafter up to 48 h p.i. (Figure 5 and Figure S5, Supporting Information). The control non‐targeted and blocking groups injected with [^89^Zr]bMSN‐PEG_5k_ and [^89^Zr]bMSN‐PEG_5k_‐TRC105 (after a blocking dose ≈50 mg kg^−1^ body weight; 6 h before injection), respectively, showed only minimal accumulation in the 4T1 tumors. Tumor uptake peaked at 3.6 ± 0.3%ID g^−1^ in the non‐targeted group and 3.0 ± 1.1%ID g^−1^ in the blocking cohorts, indicating that CD105 targeting and not only enhanced permeability and retention (EPR) effect, was responsible for the excellent and specific tumor uptake of [^89^Zr]bMSN‐PEG_5k_‐TRC105. Besides enhancement in absolute tumor uptake, [^89^Zr]bMSN‐PEG_5k_‐TRC105 administration also resulted in greater tumor contrast and more rapid detection. Tumor‐to‐muscle (T/M) ratios, as high as 47.18 ± 7.19 were obtained for the targeted group at 24 h p.i. (**Figure** **5**b and Table S6, Supporting Information). Not only T/M ratios, but also contrast index (CI) values were calculated to evaluate how well the tumor could be distinguished from normal tissues upon the introduction of targeted and non‐targeted bMSNs (Figure 5c). Distinct enhancement in the CI values further demonstrated the advantage of CD105 targeting over passive targeting alone. Both [^89^Zr]bMSN‐PEG_5k_‐TRC105 and [^89^Zr]bMSN‐PEG_5k_ nanoconjugates demonstrated long circulation time with 1.2 ± 0.3, 1.4 ± 0.8, and 1.5 ± 0.6%ID g^−1^ remaining in the blood even after 24 h p.i. in the targeted, non‐targeted, and blocking groups, respectively. Using a single bolus, two‐compartment model, the elimination half‐life (*t*_1/2β_) of [^89^Zr]bMSN‐PEG_5k_‐TRC105 was roughly evaluated to be 4.6 h (see Section S.4 and Figure S6, Supporting Information). Further studies are required for more accurate prediction of the distribution and elimination half‐lives of the intravenously injected nanoconjugates. The long residence time and enhanced tumor specific accumulation signify the promising potential of [^89^Zr]bMSN‐PEG_5k_‐TRC105 in cancer theranostics.

The uptake of the nanoconjugates in non‐target organs was confined to the RES organs, e.g., liver and spleen. Sequestration by the macrophages and phagocytes of the RES and eventual deposition in these organs is a common feature of intravenously injected nanoparticles, and was also observed in the present study. Importantly, the signal from the bone, indicative of detached ^89^Zr (^89^Zr is a well‐known osteophile^35^) remained low up to 48 h p.i. in all groups (≈1.3 ± 0.6, 1.4 ± 0.4, and 1.1 ± 0.8%ID g^−1^) and was much lower than that reported with DFO conjugated radiotracers.^24^ This finding further demonstrates the stability of intrinsic chelation of ^89^Zr by bMSNs. Interestingly, the signal from the joints increased at later time points (<8%ID g^−1^ in all groups) in accordance with the previous studies which show that ^89^Zr tends to preferentially accumulate in hypermineralized joints and epiphysis, likely due to chelation by hydroxylapatite, phosphates in the non‐soft ostium.^35^ At the terminal PET scans at 48 h p.i., mice from all three groups were euthanized and the harvested organs were analyzed by a well‐type gamma‐counter detector. Ex vivo biodistribution data validated the PET ROI results and confirmed tumor‐specific targeting with [^89^Zr]bMSN‐PEG_5k_‐TRC105 (Figure 5d).

In addition, biodistribution of the nanoparticles was studied up to day 7. A steep rise in the osteal accumulation of ^89^Zr can be clearly observed from the maximum intensity projections (MIPs) in Figure S7 (Supporting Information) at day 3, which increase persistently with time. This behavior can be attributed possibly to the degradation of the bMSN skeleton (also consistent with the in vitro studies) and to the trans‐metalation of ^89^Zr present on the bMSN exterior (more loosely coordinated than that inside) by the intrinsic chelators or plasma proteins. Interestingly, the increase in radioactivity in the joints was accompanied by a concomitant decline in the %ID g^−1^ values in the liver after day 3, likely due to the excretion of bMSNs and their fragments. Ongoing studies aim to fully explore the in vivo degradation and excretory behavior of bMSNs in complex physiological environments.

To confirm the CD105 specific uptake of the nanoconjugates, a separate cohort of 4T1 tumor‐bearing mice was used in histological analyses. Amine modified bMSNs were tagged with a fluorescent dye, fluorescein‐NHS ester (*E*_x_/*E*_m_: 495/518 nm) followed by PEGylation and TRC105 conjugation as described before. The injected mice were euthanized 6 h p.i. and tumor and other major organs were harvested and stained for CD31 (an endothelial marker). Muscle tissue was examined as a normal control. Enhanced fluorescence signal (green) from the nanoparticles can be clearly discerned in the targeted tumor tissue (Figure S8, Supporting Information), which overlapped with the signal from CD31 (red), indicating that the distribution was largely on the vasculature with minor extravasation. The non‐targeted tumor tissue, on the other hand, displays a weak signal from the nanoparticles. Consistent with our PET studies, liver and spleen tissues show high accumulation of the nanoparticles; however, the uptake is largely non‐specific with no overlap with the CD31 marker. The muscle tissue, as expected, does not emit any fluorescence signal (consistent with our PET results).

## 3 Conclusion

In summary, we report the first systematic in vivo study of intrinsically ^89^Zr‐labeled bMSNs, targeted for CD105 marker specificity in murine metastatic breast cancer model. The hierarchical, dendritic silica nanoparticles display centrally radiating mesopore channels with a two‐tiered morphology. Large, bimodal pore size (5.4 and 12 nm) distribution and the unique highly organized nanostructure of bMSNs were harnessed in this study to create a self‐destructible dual‐drug carrier. Both degradation and protein release studies demonstrated a gradual and easily tunable trend which is better suited for longer circulating nanoparticles and minimizes any premature release of the cargo. Chelator‐free ^89^Zr labeling demonstrated excellent yields and in vivo stability with marginal ^89^Zr detachment and bone uptake. Careful tailoring of the synthesis of [^89^Zr]bMSN‐PEG_5k_‐TRC105 resulted in excellent CD105 specificity, which was confirmed by extensive in vivo and ex vivo testing. Vascular targeting exhibited greater than threefold enhancement in absolute tumor uptake and normal tissue contrast, when compared with EPR dependent uptake of the nanoconjugates.

Considering the safety and easy tunability of silica nanoparticles, our simple and versatile approach to create self‐destructing angiogenesis targeted nanoplatforms can potentially pave the way for future personalized nanotheranostics. Through simple surface modifications, the nanoparticles can be tailored to (i) label a wide plethora of clinically relevant diagnostic and therapeutic radioisotopes (such as ^45^Ti (*t*_1/2_ = 3.1 h), ^177^Lu (*t*_1/2_ = 6.7 d), etc.) without the need for tiresome specific chelator chemistries, (ii) carry small‐molecule and large biomolecular drugs concurrently for combination therapy, (iii) specifically target multiple tumor types by modifying the targeting ligand, and (iv) self‐destruct to allow excretion from the body within a reasonable time period.

## 4 Experimental Section

*Materials*: TRC105 was provided by TRACON Pharmaceuticals Inc. (San Diego, CA). NHS fluorescein, Chelex 100 resin (50‐100 mesh), TEOS, triethanolamine (TEA), (3‐aminopropyl) triethoxysilane (APS), cetyltrimethylammonium chloride solution (CTAC, 25 wt%), cyclohexane, and BSA were purchased from Sigma‐Aldrich (St. Louis, MO). AlexaFluor488‐ and Cy3‐labeled secondary antibodies were purchased from Jackson Immunoresearch Laboratories, Inc. (West Grove, CA). PD‐10 columns were procured from GE Healthcare (Piscataway, NJ). Absolute ethanol, sodium chloride (NaCl), and SUN were purchased from Fisher Scientific. SCM‐PEG_5k_‐Mal was obtained from Creative PEGworks. Water and all buffers were of Millipore grade and pretreated with Chelex 100 resin to ensure that the aqueous solution was free of heavy metals. All chemicals were used as received without further purification.

*Synthesis of Biodegradable Mesoporous Silica Nanoparticles*: bMSNs with radially arranged mesochannels were synthesized using a biphase stratification method, reported previously, with slight modifications.^15^ For the synthesis of ≈160 nm sized bMSN with pore sizes of ≈5 and 12 nm, 24 mL CTAC, 180 mg TEA, and 36 mL water were stirred continuously at 60 °C for 1 h. 1 mL TEOS in 19 mL cyclohexane solution (5 v/v%) was then added gently to the mixture and stirred (125 rpm) for 60 h. As‐formed bMSNs in the aqueous layer were collected thereafter, by high speed centrifugation, followed by washing and removal of the CTAC template by immersion in 1 wt% NaCl‐methanol solution. The samples were washed repeatedly till all CTAC was removed.

*Synthesis of Mesoporous Silica Nanoparticles*: MSNs with a hydrodynamic size of ≈115 nm and pore size of 2–3 nm were synthesized as described previously.^36^ Briefly, 2 g CTAC and 20 mg TEA were stirred in 20 mL water for 1 h at room temperature. 1 mL TEOS was then added to the mixture and stirred in a water bath at 95 °C for another 1 h. As‐synthesized MSNs were collected by high speed centrifugation and subjected to NaCl‐methanol (1 wt%) treatment to remove the CTAC template.

*Synthesis of Dense Silica Nanoparticles*: dSNs with a hydrodynamic diameter of ≈100 nm were synthesized using a modified Stöber method.^23^ 35.7 mL of absolute ethanol was mixed with 5 mL of water and 0.8 mL of ammonia and stirred for 10 min at room temperature. 1 mL TEOS was then added and the mixture was allowed to react at room temperature for 1 h. dSNs were collected by centrifugation and washed with water/ethanol to remove the residual components.

*Characterization*: TEM images were obtained on an FEI T12 microscope, operated at an accelerating voltage of 120 kV. TEM samples were prepared on carbon coated copper grids following standard protocols. Nitrogen adsorption–desorption isotherms were measured at 77 K using a Quantachrome Nova 4000*e* system. The samples were first calcined at 650 °C for 12 h to ensure the removal of organic template. Surface area was determined using the BET method. Pore size distribution data were collected by the BJH method of the desorption branch of the isotherm. DLS and zeta potential analysis were performed on Nano‐Zetasizer (Malvern Instruments Ltd.). Nanoparticle number was determined using NanoSight NS300 (Malvern Instruments Ltd.).

*Amine Modification of SNs*: Amine groups were introduced onto silica nanoparticles to facilitate further surface conjugations. As‐synthesized SNs were suspended in 20 mL absolute ethanol and reacted with APS (1 mL) at room temperature for 48 h. Thereafter, the sample was washed multiple times with ethanol to get rid of residual APS. Ninhydrin test was used to determine —NH_2_ group concentration per mL of SNs.

*In Vitro Degradation in Simulated Body Fluid*: SBF with ion concentration approximately equal to those of human blood plasma was prepared per the well documented recipe (Table S7, Supporting Information).^37^ pH was adjusted to 5.5 using HCl (1 m). In vitro degradation behaviors were studied by incubating equal amounts (≈3.5 mg) of all three types of SNs in 10 mL SBF solution (pH 5.5) with gentle stirring (350 rpm) at 37 °C. Aliquots were drawn at regular intervals, replaced by equal amount of fresh SBF solution. The extracted solutions were centrifuged at 10 000 rpm. The pellet containing intact and fragmented nanoparticles was observed under the TEM. The supernatant was subjected to MP‐AES analysis (Agilent 4200 MP‐AES, CA) to determine the concentration of the released Si ions (${\left[\mathrm{Si}\right]}_{t}^{\mathrm{Supernatant}}$).In addition, initial concentration of Si ions in the three SN samples $({\left[\mathrm{Si}\right]}_{0}^{\mathrm{SN}})\;$ as well as basal Si concentration in the SBF solution $\;({\left[\mathrm{Si}\right]}_{0}^{\mathrm{SBF}})$ were separately determined. Percentage Si accumulated in the SBF at each time‐point was determined as follows

(1) $${\left[\mathrm{Si}\right]}_{t}^{\mathrm{SBF}}=\frac{{\left[\mathrm{Si}\right]}_{t}^{\mathrm{Supernatant}}-{\left[\mathrm{Si}\right]}_{0}^{\mathrm{SBF}}}{{\left[\mathrm{Si}\right]}_{n}^{\mathrm{SN}}}\times 100$$

*Cargo Loading and Release Studies*: Small molecule anticancer drug SUN and macromolecular protein BSA (≈66.5 kDa) were used as model therapeutic agents to demonstrate dual cargo encapsulation efficacy of SNs. SUN loading was carried out by dispersing equal amounts of SNs in a 2 mg mL^−1^ solution of SUN in DMSO. The mixture was incubated at room temperature for 24 h with constant shaking, followed by centrifugation to remove any un‐encapsulated SUN. SUN concentration in the supernatant was determined based on the standard curve in DMSO at 460 nm. As prepared SN(SUN) samples were subsequently added to BSA solution (2 mg mL^−1^ in PBS) and mixed gently for 24 h. Excess unloaded BSA was removed by centrifugation and the supernatant was quantified at 280 nm using Nanodrop fluorospectrometer.

Loading efficacy was defined as the ratio of guest molecule loaded in the nanoparticles to the total amount added

(2) $$\mathrm{Loading}\;\;\mathrm{efficiency}\left(\%\right)=\frac{\mathrm{Amount}\;\;\mathrm{of}\;\;\mathrm{drug}\;\;\mathrm{loaded}\;\;\mathrm{in}\;\;\mathrm{SNs}}{\mathrm{Total}\;\;\mathrm{amount}\;\;\mathrm{of}\;\;\mathrm{drug}\;\;\mathrm{added}}\times 100$$

Loading capacity defined as amount of drugs loaded per g of nanocarrier, was calculated using the following equation

(3) $$\mathrm{Loading}\;\;\;\mathrm{capacity}\left(\%\right)=\frac{\mathrm{Amount}\;\;\mathrm{of}\;\;\mathrm{drug}\;\;\mathrm{loaded}\;\;\mathrm{in}\;\;\mathrm{SNs}}{\mathrm{Amount}\;\;\mathrm{of}\;\;\mathrm{SNs}}\times 100$$

To carry out the release study, bMSN(SUN‐BSA) and MSN(SUN‐BSA) were suspended in SBF solution (pH 5.5) accompanied with gentle shaking. Aliquots were drawn periodically, centrifuged at high speed, and tested for released SUN and BSA using UV–vis absorption at 460 and 280 nm, respectively.

*^89^Zr Production*: ^89^Zr‐oxalate was produced according to previously reported procedures by the University of Wisconsin–Madison cyclotron group.^38^ Briefly, natural yttrium‐89 (^89^Y) foil (250 μm, 99.9%) was irradiated with a proton beam to produce ^89^Zr via the ^89^Y(p,n)^89^Zr reaction by using a 16 MeV GE PETtrace cyclotron. After isotope separation and purification, ^89^Zr‐oxalate was obtained, with a specific activity of >20 GBq μmol^−1^ of ^89^Zr.

*Chelator‐Free ^89^Zr Labeling*: 1 mL of as‐prepared aminated SNs in HEPES buffer (pH 7.5; 0.1 m) were mixed with 111 MBq ^89^Zr oxalate and the pH was adjusted to 8–9 using Na_2_CO_3_ solution (2 m). The mixture was incubated at 75 °C for 4 h. Thin layer chromatography (TLC) was used to quantify ^89^Zr labeling yields at different time points, using 0.05 m EDTA as the mobile phase. Final labeled products were collected by centrifugation.

*In Vitro Serum Stability*: In vitro stability of the isotope was evaluated in serum by incubating 300 μL of [^89^Zr]SN with 300 μL of 2× whole mouse serum with constant shaking at 37 °C for 7 d. At each time‐point, 100 μL of the sample was withdrawn and purified by a 100 kDa centrifugal filter. Radioactivity in the filtrate and retentate were measured using a gamma counter and decayed back to day 0 for further calculations.

*Thiolation of TRC105*: TRC105 antibody was thiolated to facilitate conjugation to bMSNs. 100 μL of TRC105 (7 mg mL^−1^) was mixed with Traut's reagent (2 mg mL^−1^) in ≈1:30 molar ratio and reacted at room temperature for 1 h. The pH of the solution was adjusted to 8, using 0.1 m Na_2_CO_3_. At the end of the incubation period, the mixture was purified on PD‐10 columns to get rid of residual Traut's reagent. The final concentration of TRC105‐SH was quantified using Nanodrop instrument. Ellman's reagent was used to quantify the number of —SH moieties per antibody.

*Synthesis of [^89^Zr]bMSN‐PEG_5k_‐TRC105*: [^89^Zr]bMSN‐NH_2_ was further surface modified to make them suitable for in vivo targeted imaging. [^89^Zr]bMSN‐NH_2_ water solution (pH 6–7) was added to 2.5 mg (500 nmol) SCM‐PEG_5k_‐Mal and reacted for 1 h at room temperature. Afterward, the excess PEG was removed by centrifugation. The PEGylation step was repeated under similar conditions. [^89^Zr]bMSN‐PEG_5k_ was obtained after washing atleast twice with PBS and divided into two parts. Finally, 2.5 nmol of TRC105‐SH was added to one‐half of the above solution and incubated at room temperature for 1 h with gentle shaking. [^89^Zr]bMSN‐PEG_5k_‐TRC105 was obtained as the final product and used for the targeted and blocking groups. The other half ([^89^Zr]bMSN‐PEG_5k_) was used as the non‐targeted group.

*4T1 Tumor Models*: 4T1 breast cancer cells were cultured in RPMI 1640 medium (supplemented with FBS and antibiotics). 2 × 10^6^ cells, suspended in PBS were injected subcutaneously into the front flank of 4–5 weeks old Balb/c mice (Envigo, IN, USA) to establish the tumors. The animals were used for in vivo experiments once the tumor diameters reached 5–8 mm.

*In Vivo Vasculature Targeted PET Imaging*: All animal studies were conducted per the protocol approved by the University of Wisconsin Institutional Animal Care and Use Committee. 4T1 tumor‐bearing mice were randomly divided into three groups (*n* = 3). 150 μL (≈7.4 MBq) of either [^89^Zr]bMSN‐PEG_5k_‐TRC105 or [^89^Zr]bMSN‐PEG_5k_ was injected intravenously and serial PET scans were performed at various time‐points. The mice in the blocking group were each injected with unlabeled TRC105 antibody (dose: 5 mg kg^−1^), 6 h before [^89^Zr]bMSN‐PEG_5k_‐TRC105 administration.

Serial PET scans (at 0.5, 6, 24, and 48 h p.i.) were performed using a microPET/microCT Inveon rodent model scanner (Siemens Medical Solutions USA, Inc.). Image acquisition, reconstruction, and ROI analysis were performed as described previously.^38^ All PET data were decay corrected to the initial time‐point and is presented as percentage injected dose per gram of tissue (%ID g^−1^). Contrast enhancement was calculated in terms of two parameters: Tumor‐to‐muscle ratio

(4) $$\left(\mathrm{T/M}\right)=\frac{{\mathrm{BqmL}}^{-1}\;\;\mathrm{in}\;\;\mathrm{tumor}}{{\mathrm{BqmL}}^{-1}\;\;\mathrm{in}\;\;\mathrm{muscle}}$$

(5) $$\mathrm{Contrast}\;\;\;\mathrm{index}\;\;\;\left(\mathrm{CI}\right)=\frac{{\mathrm{BqmL}}^{-1}\;\left(\mathrm{tumor}\right)\;-\;{\mathrm{BqmL}}^{-1}\;\left(\mathrm{muscle}\right)}{{\mathrm{BqmL}}^{-1}\;\left(\begin{array}{c} \mathrm{contralateral}\\ \mathrm{region} \end{array}\right)\;-\;{\mathrm{BqmL}}^{-1}\;\left(\mathrm{muscle}\right)}$$

(Same volume ROIs were drawn on tumor and contralateral regions on the same plane/frame in the tomographic dataset.)

*Ex Vivo Biodistribution*: Ex vivo biodistribution studies were carried out after the final PET scans at 48 h p.i. to ensure that the quantitative uptake from the PET ROI values truly represented the radioactivity distribution in 4T1 tumor‐bearing mice. The mice were euthanized and all the major tissues were harvested and wet weighted. The radioactivity measurements for each tissue were carried out using a gamma counter (Perkin Elmer, MA, USA) and presented as %ID g^−1^.

*Histology*: To carry out histological analysis of the major tissues, fluorescein‐NHS dye conjugated bMSNs (FITC‐bMSN‐PEG_5k_‐TRC105 and FITC‐bMSN‐PEG_5k_) were injected intravenously in 4T1 tumor‐bearing mice. The tissues were harvested at 6 h p.i. and fixed in optimum cutting temperature (OCT) formulation prior to sectioning. Frozen tissue slices of 6 μm were fixed with cold acetone and stained for endothelial marker CD31, as described previously through the use of a rat anti‐mouse CD31 antibody and a Cy3‐labeled donkey anti‐rat IgG.^39^ All images were acquired with a Nikon Eclipse Ti microscope.

## Supporting information

As a service to our authors and readers, this journal provides supporting information supplied by the authors. Such materials are peer reviewed and may be re‐organized for online delivery, but are not copy‐edited or typeset. Technical support issues arising from supporting information (other than missing files) should be addressed to the authors.

Supplementary

Supplementary
