# Supplementary material for: Engineering Intrinsically Zirconium‐89 Radiolabeled Self‐Destructing Mesoporous Silica Nanostructures for In Vivo Biodistribution and Tumor Targeting Studies
Source: Adv Sci (Weinh). 2016 May 27;3(11):1600122. doi: 10.1002/advs.201600122 (PMC5102673; doi:10.1002/advs.201600122)
Supplement: Supplementary file 1 — Supplementary [file ADVS-3-0a-s001.pdf]

Copyright WILEY-VCH Verlag GmbH & Co. KGaA, 69469 Weinheim, Germany, 2016.

ADVANCED SCIENCE
Open Access

Supporting Information

for *Adv. Sci.,* DOI: 10.1002/advs.201600122

Engineering Intrinsically Zirconium-89 Radiolabeled Self-Destructing Mesoporous Silica Nanostructures for In Vivo Biodistribution and Tumor Targeting Studies

*Shreya Goel, Feng Chen, Shijie Luan, Hector F. Valdovinos, Sixiang Shi, Stephen A. Graves, Fanrong Ai, Todd E. Barnhart, Charles P. Theuer, and Weibo Cai**

Copyright WILEY-VCH Verlag GmbH & Co. KGaA, 69469 Weinheim, Germany, 2013.

Supporting Information

Engineering Intrinsically Zirconium-89 Radiolabeled Self-Destructing Mesoporous Silica Nanostructures for In Vivo Biodistribution and Tumor Targeting Studies

*Shreya Goel, Feng Chen, Shijie Luan, Hector F. Valdovinos, Sixiang Shi, Stephen A. Graves, Fanrong Ai, Todd E. Barnhart, Charles P.Theuer, Weibo Cai**

S. Goel and S. Shi
Materials Science Program, University of Wisconsin – Madison, Wisconsin 53705

Dr. F. Chen and Dr. F. Ai
Department of Radiology, University of Wisconsin – Madison, Wisconsin 53705

S. Luan
School of Pharmacy, Temple University, Philadelphia, Pennsylvania 19140

H.F. Valdovinos, S. Graves and Dr. T.E. Barnhart
Department of Medical Physics, University of Wisconsin – Madison

Dr. C.P. Theuer
TRACON Pharmaceuticals Inc., San Diego, California 92122

Prof. W. Cai
Materials Science Program, University of Wisconsin – Madison, Wisconsin 53705
Department of Radiology, University of Wisconsin – Madison, Wisconsin 53705
Department of Medical Physics, University of Wisconsin – Madison
University of Wisconsin Carbone Cancer Centre, Madison, Wisconsin 53705
Fax: (+1) 608-265-0614
Email: WCai@uwhealth.org
Homepage: http://mi.wisc.edu

Keywords: Biodegradable mesoporous silica nanoparticles, Intrinsic radiolabeling, Positron emission tomography (PET) imaging, Vasculature targeting

S.1. Theoretical Calculations for the number of nanoparticles (bMSNs)

Theoretical calculations to determine the number of nanoparticles per gram were based on a previously reported procedure.[1]

Volume of a mesoporous nanoparticle, such as bMSN can be divided into (i) pore volume, (V_p) and solid volume (V_s). V_p was determined from the BET data, while Vs was determined using the following equation: $Vs = \frac{M_s}{\rho_s} = \frac{1}{2.2} = 0.455\ cm^3$ where Ms is the mass of bMSNs in grams and ρ is the density of silica (2.2 gcm^{-3}).

Number of bMSNs per gram $N_{bMSN} = \frac{1}{M_{bMSN}}$, where M_{bMSN} is the mass of each nanoparticle.

$M_{bMSN} = \rho * V_{bMSN} = \rho * \frac{4\pi r^3}{3}(1-\chi)$; where r = radius of bMSN as determined from TEM images (*Figure S2c*; r = 82.68 nm) and χ is the pore volume fraction defined as $\chi = \frac{Vp}{Vp+Vs}$. Therefore, making the appropriate substitutions, number of bMSNs per gram can be estimated to be:

$$N_{bMSN} = \frac{1}{\rho * \frac{4\pi r^3}{3}(1-\frac{Vp}{Vp+Vs})} = \frac{0.749\ (Vp+0.455)}{\pi r^3} = \frac{0.238\ (1.844+0.455)}{(82.68 \times 10^{-7})^3} = \mathbf{9.68 \times 10^{14}}$$

The theoretically calculated number was found to be very close to that determined using the NanoSight Technology ~**8.97 × 10^{11} bMSNs mL^{-1}** or **3.59× 10^{14} g^{-1}**.

S.2. Calculation of the number of silanol groups per bMSN

According to the Zhuravlev model, the number of silanol groups (Si-OH) per unit surface area of amorphous silica is depicted by a physico-chemical constant (known as the Kiselev–Zhuravlev constant) with a numerical value ~4.9 nm^{-2}.[2] The number of silanol groups for bMSN (5 v/v%), thus calculated is presented in *Table S1*.

S.3. Calculation of the number of targeting ligands (TRC105) per bMSN

Prior to conjugation, TRC105 was derivatized with Traut's Reagent, such that each antibody molecule possessed ~3 –SH moieties (as determined by the Ellman's Reagent test).[3] Initial amount of TRC105-SH added to the nanoparticle solution = 45.80 µg or 1.83×10^{14} molecules. After incubation with bMSN-PEG_{5k}-Mal (~8.97×10^{11} mL^{-1}) for 3 h, the solution was centrifuged at 5000g to remove any unconjugated antibody. Amount of unreacted TRC105-SH in the supernatant was calculated from the Bradford Assay using the standard curve after polynomial fitting (*Figure S4*). Amount of unreacted TRC105-SH was determined to be ~35.82 µg. Thus, ~4.02×10^{13} molecules of TRC105-SH were finally conjugated to the bMSN-PEG_{5k}-Mal. Considering the number of nanoparticles per reaction to be ~8.97×10^{11} mL^{-1}, the number of TRC105-SH conjugated per nanoparticle per mL was determined to be **~44.6**.

S.4. Prediction of elimination half-life ($t_{1/2\beta}$) of [^{89}Zr]bMSN-PEG_{5k}-TRC105

Elimination half-life ($t_{1/2\beta}$) of [^{89}Zr]bMSN-PEG_{5k}-TRC105 was roughly estimated using the quantification data obtained from serial PET scans in 4T1 tumor bearing mice (n = 4). Equal volume regions-of-interest (ROIs) were drawn over the heart tissue in the PET images and used to calculate percentage injected dose per gram of tissue (%ID/g) at each time-point. All data was decay corrected and processed as previously described.[4] Bi-exponential fitting of the time-activity curve was used to estimate $t_{1/2\beta}$ of [^{89}Zr]bMSN-PEG_{5k}-TRC105 (*Figure S7*).

SUPPLEMENTARY TABLES AND FIGURES

Table S1. Number of silanol groups per nanoparticle

	Radius (nm)	Surface Area (m^2/g)	No. of Nanoparticles per gram	Surface Area per Nanoparticle (nm^2)	Total Number of Silanol Groups per Nanoparticle
**bMSN(5 v/v%)**	82.68	741.92	3.59×10^{14}	2.06×10^{6}	1.01×10^{7}

Table S2. Sunitinib (SUN) loading and release

	Amount Loaded (mg)	Loading Capacity (mg/g)	Loading Efficiency (%)	Cumulative Release (%)
**dSN**	0.17	46.63	8.39	-
**MSN**	0.56	155.09	27.9	56.41
**bMSN**	0.83	295.95	41.4	68.90

Table S3. Bovine Serum Albumin (BSA) loading and release

	Amount Loaded (mg)	Loading Capacity (mg/g)	Loading Efficiency (%)	Cumulative Release (%)
**dSN**	0.02	20.00	1.5	-
**MSN**	0.15	250.00	15.0	80.0
**bMSN**	0.33	589.28	32.5	90.77

Table S4. Temporal variation in labeling yields of bMSN-NH_2 in 0.1 M HEPES buffer at 75 °C under different pH conditions

	**pH 2**	**pH 7**
**30 min**	19.1	67.2
**60 min**	28.1	88.5
**120 min**	30.7	94.7

Table S5. Change in zeta potential during the stepwise synthesis of [^{89}Zr]bMSN-PEG$_{5k}$-TRC105. All measurements were performed in PBS (pH 7.4)

	**Zeta Potential (mV)**
**bMSN**	-48.37 ± 0.31
**bMSN-NH_2**	9.58 ± 0.92
**bMSN-PEG$_{5k}$**	-2.78 ± 0.33
**bMSN-PEG$_{5k}$-TRC105**	-1.28 ± 0.39
**[^{89}Zr]bMSN-PEG$_{5k}$-TRC105**	-0.16 ± 0.06

Table S6. Tumor-to-muscle ratios calculated based on the PET ROI values (n=3)

	**Targeted**	**Non-targeted**	**Blocking**
**0.5 h**	7.49 ± 2.55	2.16 ± 0.68	3.39 ± 0.64
**4 h**	25.89 ± 3.55	5.04 ± 0.85	5.74 ± 0.17
**24 h**	47.18 ± 7.19	9.75 ± 2.07	9.92 ± 2.82
**48 h**	47.19 ± 8.41	7.95 ± 1.64	8.84 ± 2.32

Table S7. Simulated Body Fluid (SBF) composition (pH 7.4; 1 L)

Reagent	Amount
NaCl	7.996 g
$NaHCO_3$	0.350 g
KCl	0.224 g
$K_2HPO_4 \cdot 3H_2O$	0.228 g
$MgCl_2 \cdot 6H_2O$	0.305 g
1M-HCl	40 mL
$CaCl_2$	0.278 g
Na_2SO_4	0.071 g
$(CH_2OH)_3CNH_2$	6.057 g

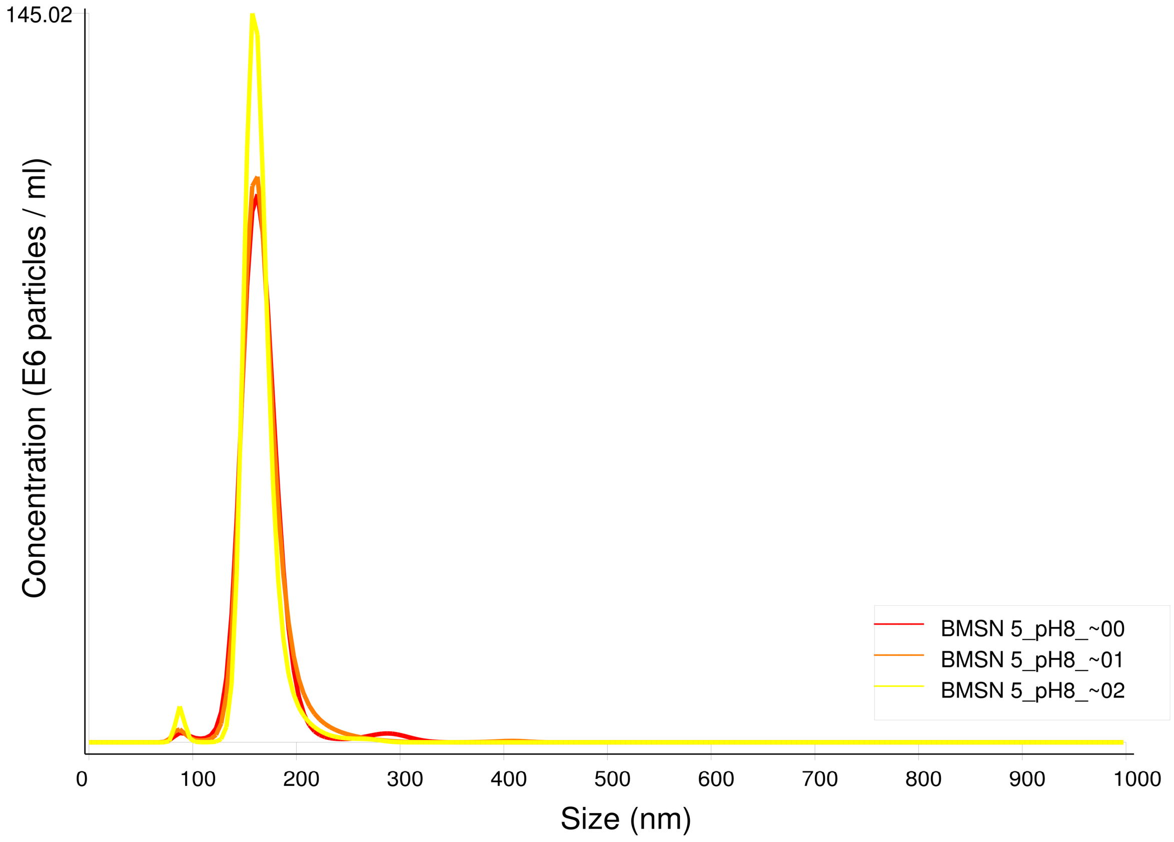

Figure S1. Size-Concentration graph for 1000-times diluted solution of bMSN (5 v/v%).

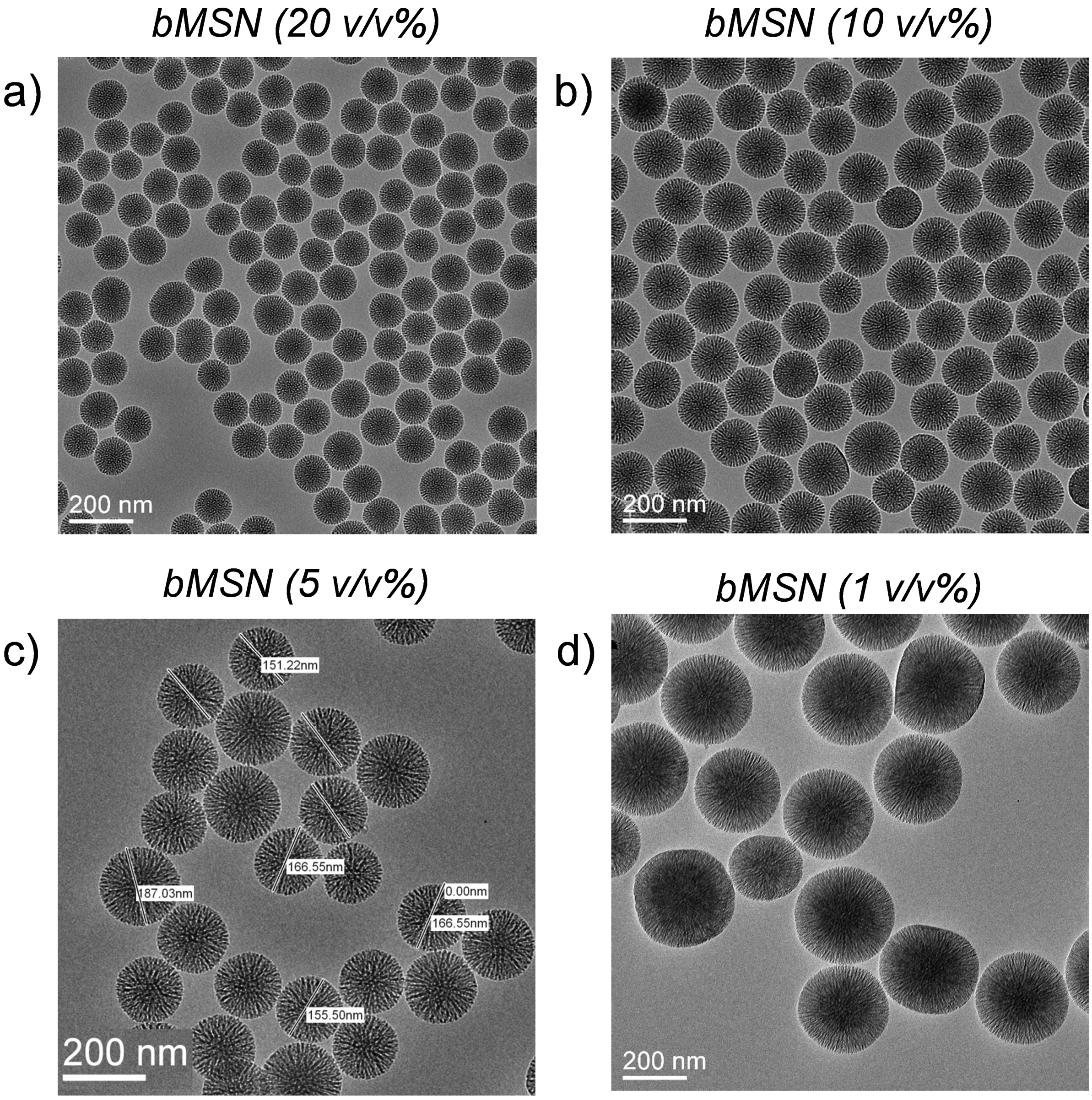

Figure S2. TEM images of bMSNs with varying compositions (TEOS: cyclohexane ratios) and pore sizes. (a) 20 v/v % (b) 10 v/v %, (c) 5 v/v % and (d) 1 v/v%

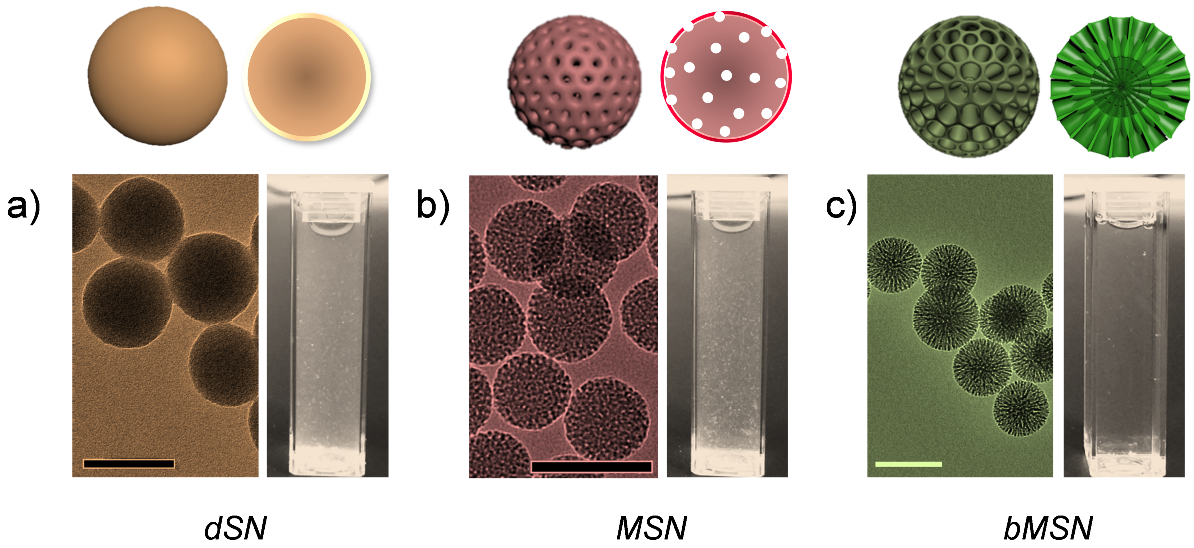

Figure S3. (**a**) TEM image of dSN (left) and a photograph of dSN solution after 21 days of incubation in SBF. (**b**) TEM image of MSN (left) and a photograph of MSN solution after 21 days of incubation in SBF. (**c**) TEM image of bMSN (left) and a photograph of bMSN solution after 21 days of incubation in SBF. Schematic on the top depicts the 3-D morphology and 2-D cross-sections of the corresponding nanoparticles.

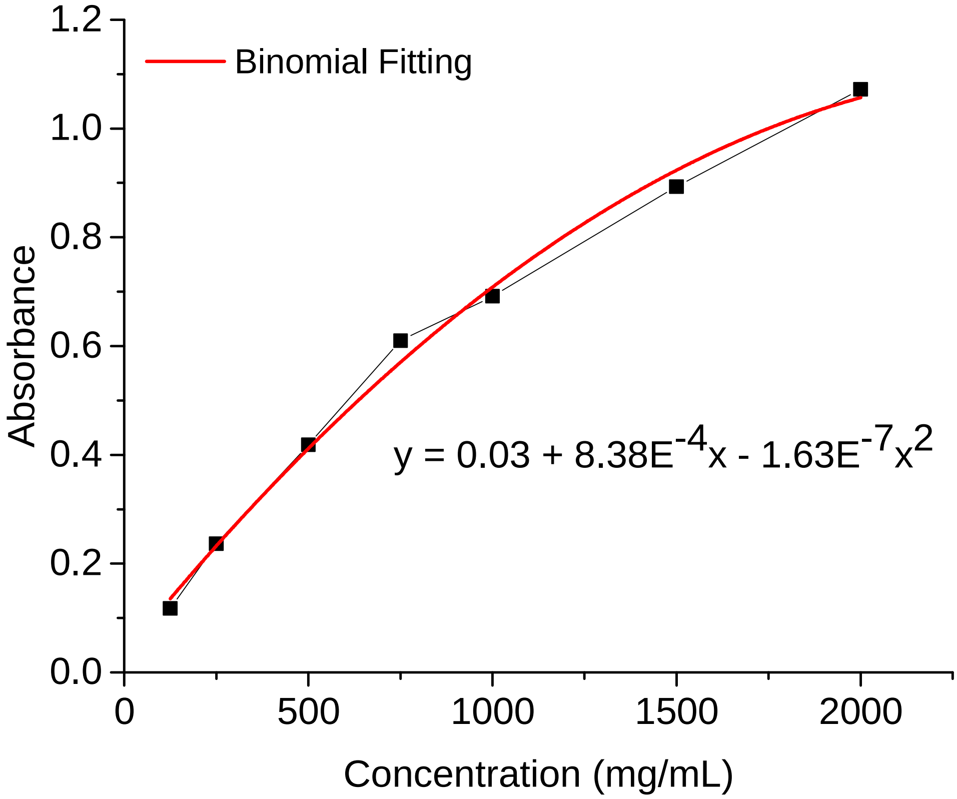

Figure S4. Equation and standard curve from Bradford Assay for calculation of number of TRC105 per bMSN.

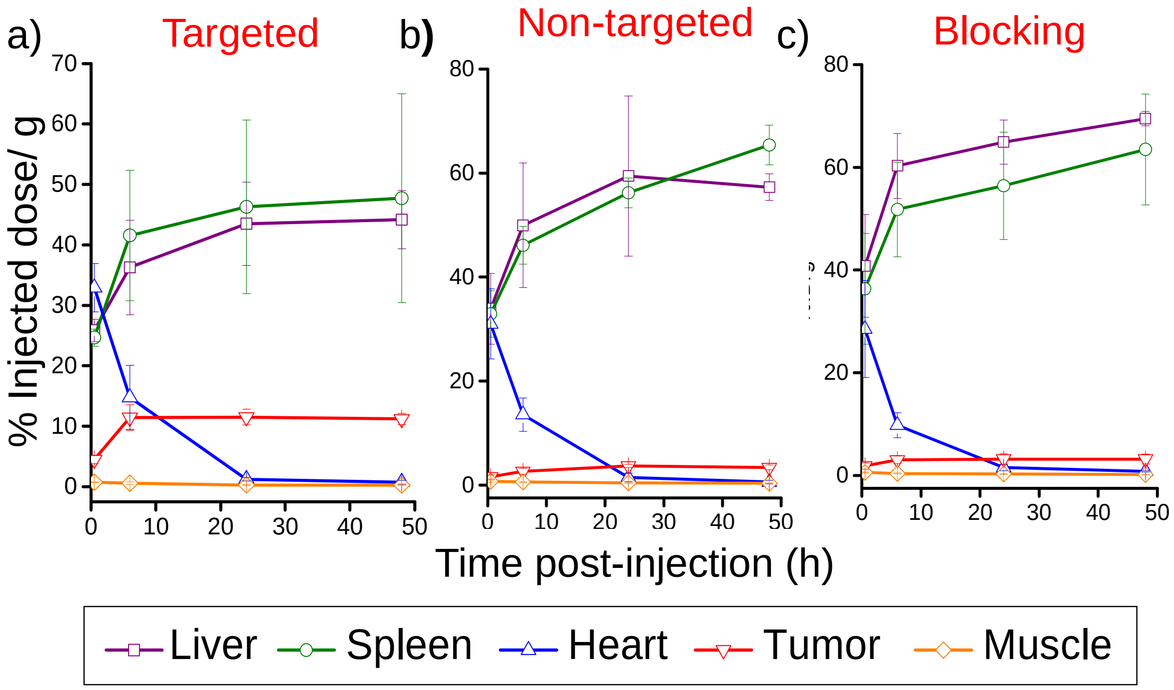

Figure S5. Region-of-interest (ROI) quantification. Time-activity curves for (**a**) targeted, (**b**) non-targeted, and (**c**) blocking groups.

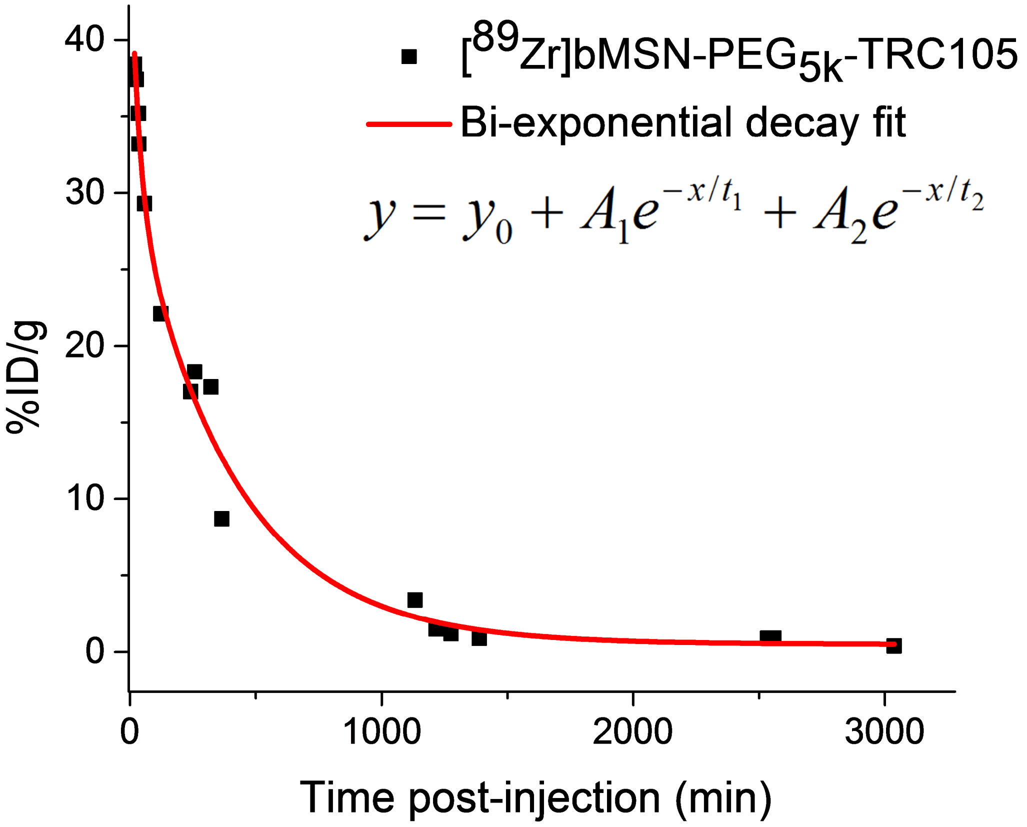

Figure S6. Bi-exponential fitting of the time-activity curve to determine the elimination half-life ($t_{1/2\beta}$) for [^{89}Zr]bMSN-PEG$_{5k}$-TRC105.

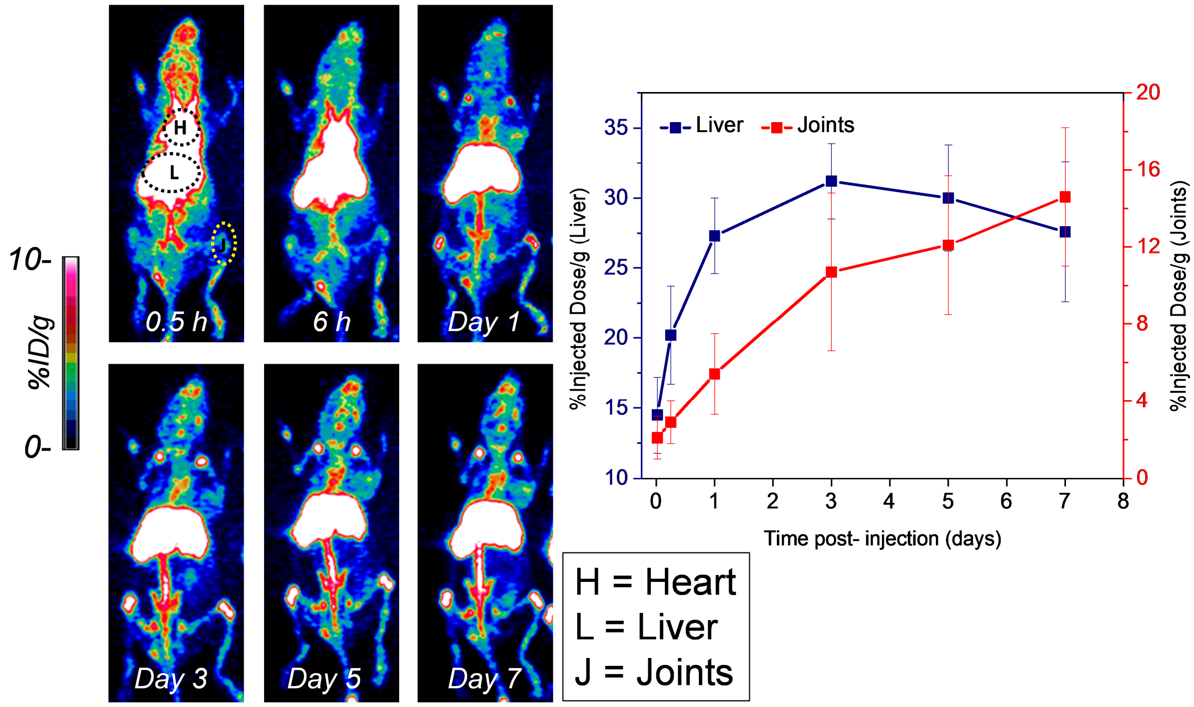

Figure S7. (**a**) Maximum-intensity-projections (MIP) of long term fate of [^{89}Zr]bMSN-PEG$_{5k}$-TRC105 in healthy mice.

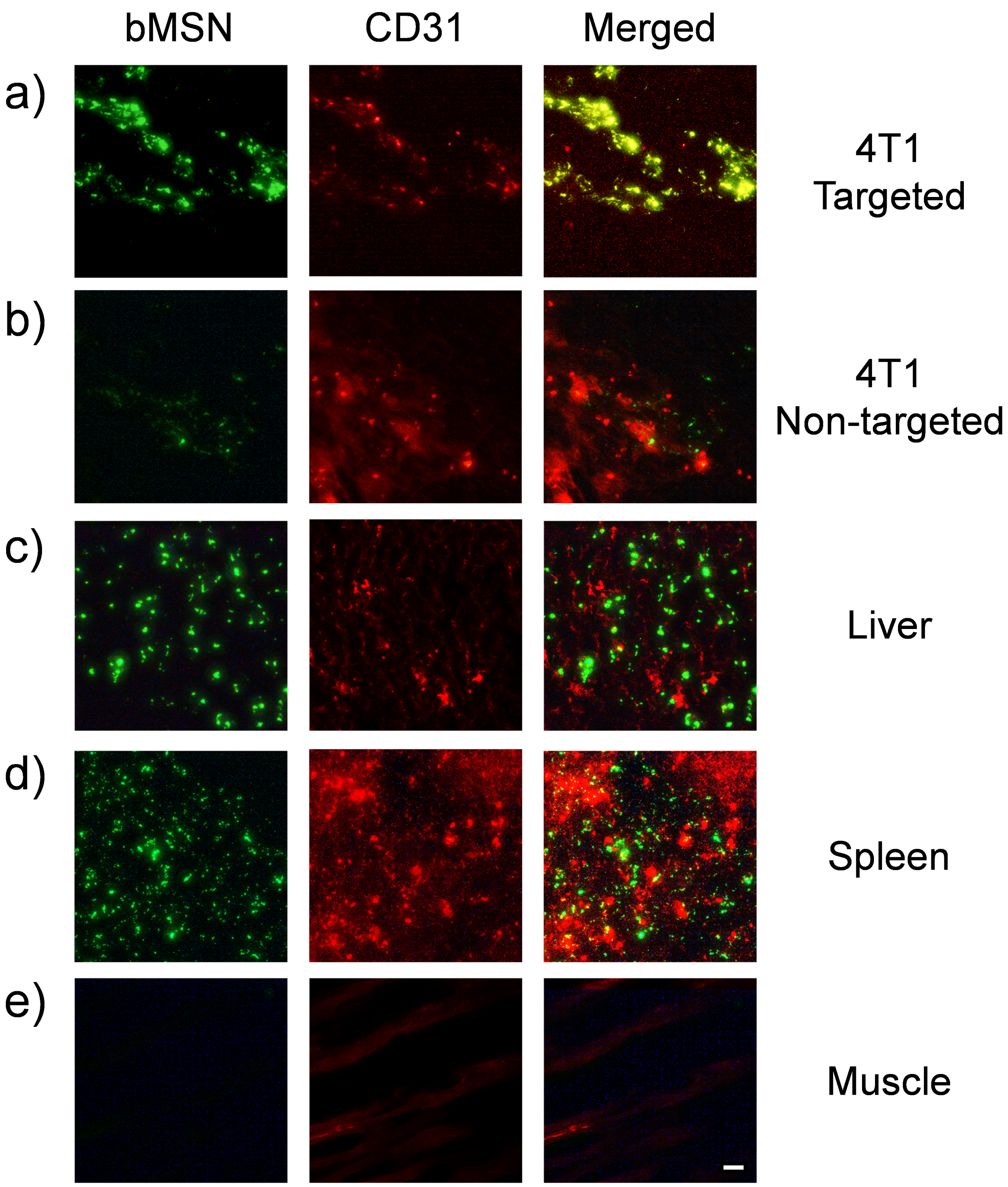

Figure S8. Ex vivo histology 6 h p.i. Fluorescein conjugated bMSNs are shown in green, (left panel) and immuno-stained CD31 is shown in red (middle panel). Merged images are also shown. Scale bar presents 50 µm.

References

[1] L. Pan, Q. He, J. Liu, Y. Chen, M. Ma, L. Zhang, J. Shi, *J Am Chem Soc* 2012, 134, 5722.

[2] Y. S. Lin, C. L. Haynes, *J Am Chem Soc* 2010, 132, 4834.

[3] L. T. Zhuravlev, *Colloids Surf A* 2000, 173, 1.

[4] J. Woodward, J. Tate, P. C. Herrmann, B. R. Evans, *J Biochem Biophys Methods* 1993, 26, 121.

[5] H. Hong, G. W. Severin, Y. Yang, J. W. Engle, Y. Zhang, T. E. Barnhart, G. Liu, B. R. Leigh, R. J. Nickles, W. Cai, *Eur J Nuc Med Mol Imaging* 2012, 39, 138.
